# *5-HTT* genotype and inertia of negative affect in adolescents and young adults from the general population

**DOI:** 10.1007/s00702-022-02459-8

**Published:** 2022-03-04

**Authors:** T. M. Ollmann, E. Seidl, J. Venz, L. Pieper, C. Voss, J. Hoyer, H. Kische, S. R. Poppenhäger, M. A. Schiele, K. Domschke, K. Beesdo-Baum

**Affiliations:** 1 Behavioral Epidemiology, Institute of Clinical Psychology and Psychotherapy, Technische Universität Dresden, Chemnitzer Str. 46, 01187 Dresden, Germany; 2 Center for Clinical Epidemiology and Longitudinal Studies (CELOS), Institute of Clinical Psychology and Psychotherapy, Technische Universität Dresden, Dresden, Germany; 3 Department of Psychiatry and Psychotherapy, Medical Center, Faculty of Medicine, University of Freiburg, Freiburg, Germany; 4 Center for Basics in NeuroModulation, Medical Faculty, University of Freiburg, Freiburg, Germany

**Keywords:** Emotional inertia, *5-HTT*LPR, Negative affect, Adolescence

## Abstract

The study aims to replicate the previous found association of *5-HTT*LPR and inertia of negative affect in daily life of adolescents and young adults. Data of 877 adolescents (aged 14–21 years) of the Behavior and Mind Health (BeMIND) study (epidemiological cohort study, Dresden, Germany) were genotyped for 5-*HTT*LPR/rs25531, grouped into SS/SL_G_/SL_A_/L_G_L_A_/L_G_L_G_ vs. L_A_L_A_, and provided ratings on negative affect items, depression and anxiety (Patient-Reported Outcomes Measurement Information System) eight times a day over 4 days. Multilevel regression models did not reveal an association of 5-*HTT*LPR genotype and inertia of negative affect, nor associations with inertia of anxiety or depression. Inertia of negative affect seems not to be a psychological mechanism through which *5-HTT*LPR acts on psychopathology.

## Introduction

Change in dynamics of affective experience has gathered increasing attention within the last years since the static view of emotional experiences ignores the flow of affect influenced by internal and external factors. Fluctuations of emotions have been suggested to be associated with aspects of psychological wellbeing and maladaptation (Houben et al. 2015) as well as mental disorders (Trull et al. 2015). Thus, detecting and analyzing dynamic patterns of affective experience is crucial to improve our understanding of mental health problems.

The use of smartphone-based Ecological Momentary Assessment (EMA) has recently improved the assessment of such dynamic processes. Various measures have been proposed to depict different dynamical aspects of everyday life experiences (Dejonckheere et al. 2019). One frequently used measure is emotional inertia, which has previously been linked to mental health problems (Houben et al. 2015; Wichers et al. 2015). Emotional inertia stands for the resistance to change in affect over time and is usually calculated as the autoregressive coefficient (Lamers et al. 2018; Trull et al. 2015; Wichers et al. 2015).

Therefore, high emotional inertia is present when current emotional states are heavily influenced by previous states and low emotional inertia is present when current feelings are less predictable by previous feelings. Thus higher levels of emotional inertia might point towards the decouplement of emotional states from their adaptive function to respond flexibly to significant environmental events and regulation efforts to make disruptive emotions return to baseline (van Roekel et al. 2018).

Recently, emotional inertia has been associated with several mental health related constructs including low self-esteem (Koval et al. 2012; Kuppens et al. 2010), rumination (Koval et al. 2012; Trull et al. 2015) and depression (Koval et al. 2012; Kuppens et al. 2010). Higher levels of emotional inertia have been found in depressed adolescents compared to non-depressed adolescents (Koval et al. 2012; Kuppens et al. 2010). Furthermore, higher levels of emotional inertia predicted the onset of depression in early adolescents in a longitudinal study two years later (Kuppens et al. 2012), as well as depression severity in a non-clinical adult and clinical adolescent sample (Koval et al. 2012). Based on these findings, van Roekel et al. (2018) pursued the assumption that emotional inertia may be rooted in a known genetic risk factor for emotional dysfunction, namely a length polymorphism in the regulatory region of the serotonin transporter gene (serotonin transporter gene linked polymorphic region, *5-HTT*LPR). *5-HTT*LPR comprises a short allele (S) and a long allele (L) whereas the short allele is associated with less transcription of the serotonin transporter compared with the long allele (Lesch et al. 1996). A single nucleotide polymorphism rs25531 (A > G) is assumed to render the L_G_ allele functionally equivalent to the S allele (i.e. reduced 5-HTT availability).

In their study, van Roekel et al. (2018) conducted an experience sampling study in the Netherlands among 236 high school adolescents investigating the association of 5*-HTT*LPR/rs25531 and emotional inertia and discerned S-allele carriers to be characterized by higher inertia for negative affect. The negative affect was operationalized by calculating a mean negative score using different emotional and affective states, including feeling anxious, irritated, worried, low, insecure, and guilty. The association between emotional inertia regarding negative affect and *5-HTT*LPR/rs25531 was also found after adjusting for age, gender and depressive symptoms. The authors concluded that emotional inertia may represent a possible psychological pathway how *5-HTT*LPR contributes to risk for depression or more broadly affective disorders. Following these results, the aim of the present study was to replicate this finding by van Roekel et al. (2018) using a larger population sample of adolescents and young adults.

## Methods

### Sample and procedures

The data were taken from the baseline investigation of the Behavior and Mind Health (BeMIND) study, an epidemiological cohort study of adolescents and young adults from Dresden, Germany. A random age and sex stratified general population sample of 14–21 year olds was drawn from the population registry in 2015 and *N* = 1180 participated in the baseline investigation conducted from 11/2015 to 12/2016 (response proportion: 21.7%; AAPOR formula RR1; cooperation rate: 43.4%; AAPOR formula coop1; AAPOR 2016). The overall aim of the study is to investigate developmental trajectories and risk factors of mental and behavioral disorders. Baseline assessments included categorical and dimensional diagnostic assessment on day 1, cognitive tasks and biosampling (blood or buccal swab) at day 2 approximately one week later, and an online questionnaire assessment as well as an Ecological Momentary Assessment (EMA) study part (see Appendix, Table 4) in between these two personal appointments. For the present analysis, participants were excluded if they did not participate in the EMA assessment (*N* = 26), if they showed a compliance rate of < 50% in EMA (no reliable and sufficient data; *N* = 82), if they did not provide blood/buccal swab samples/no genotype information available (*N* = 101), if they had no Caucasian descent by first generation (*N* = 115) or if information about the descent were not available (*N* = 35), resulting in a final analysis sample of 877 participants (74.2% of the total sample). Note that the numbers do not sum up since some participants fall under more than one category. All participants gave written informed consent or assent (in minors also legal guardians provided written informed consent). The study protocol and its amendments were reviewed and accepted by the ethics committee of the Technische Universität Dresden, Germany (TUD: EK381102014) and the study was conducted in accordance with the ethical standards laid down in the 1964 Declaration of Helsinki and its later amendments. Detailed information on the studies aims, procedures and sample characteristics can be found elsewhere (Beesdo-Baum et al. 2020).

### Ecological momentary assessment

On eight occasions per day over four consecutive days (2 week-days and the weekend), smartphone-based EMA assessments were administered. Daily assessments included a time-based morning assessment, six daytime assessments, and one evening assessment. All items used for the following analyses were mandatorily assessed at each of the assessments, using a seek-bar which translated into a scale of 0–100. Sleep times and periods during which the participants did not want to be disturbed (e.g. school times) were considered while setting up an individual reminder scheme. Assessments were distributed symmetrically throughout the day but at unknown points of time for the participant. Each survey could be postponed three times for 5 min (15 min altogether), or the questionnaire could be omitted. To enhance the motivation for the execution of the EMA, participants were instructed face to face by trained study staff and a training day with three sets of questionnaires was taking place beforehand. Smartphones were returned to the study center by the participants and data stored on the smartphone was then transferred to the study server.

For the purpose of replicating the findings by van Roekel et al. (2018), a negative affect score was generated by calculating the mean of emotional/affect items and scales used in the EMA assessment. As van Roekel et al. (2018) did not use a validated negative affect scale, but added up items assessing negative affect, namely feelings of anxiety, irritation, worry, low mood, insecurity and guilt, the present study followed this procedure. However, the items differ as the current study used items of negative valence of the BeMIND EMA-item pool, namely anxiety (Patient-Reported Outcome Measurement Information System Version 1.0 Short Form; PROMIS-ANX; Pilkonis et al. 2011), depression (Patient-Reported Outcomes Measurement Information System Version 1.0 Short Form; PROMIS-DEP; Pilkonis et al. 2011), anger (Patient-Reported Outcomes Measurement Information System Version 1.0 Short Form; PROMIS-DEP; Pilkonis et al. 2011), wakefulness (short-form of the Multidimensional mental-state questionnaire; MDBF; Wilhelm and Schoebi 2007), pessimism (Skala Optimismus-Pessimismus-2; SOP2; Kemper et al. 2012), negative thoughts, and experiential avoidance (self-developed; full descriptions of the items used in this study are presented in Table 4 in the Appendix).

### Genotyping

EDTA-blood samples were stored without delay at − 80 °C in a laboratory freezer. Whenever participants (or legal guardians) did not provide consent/assent to draw blood, they were asked to provide a buccal swab sample. The final analysis sample was genotyped for the *5-HTT*LPR as well as the functionally related single nucleotide polymorphism rs25531 as described in published protocols (for details see Schiele et al. 2016). Genotypes were determined by two independent blinded investigators. Hardy–Weinberg criteria were fulfilled for the *5-HTT*LPR genotype distribution (SS = 119, SL = 408, LL = 350,* p* = 0.99) as well as for the triallelic model (L_A_L_A_ = 289, L_G_L_A_/SL_A_ = 421, L_G_L_G_/SL_G_/SS = 167, *p* = 0.53).

### Statistical analysis

We applied sample weights to make sure that, after a weighting adjustment for sex and age, the age/sex distribution of the sample was representative for the population of the 14–21-year-old participants of Dresden. Two groups were built comprising L_A_L_A_ carriers in the high-expression group and SS, SL_G_, SL_A_, L_G_L_A_, L_G_L_G_ carriers in the low-expression group (Baffa et al. 2010; Baune et al. 2008; Schiele et al. 2016, 2020; Wendland et al. 2006). A group comparison between the low-and high-expression group was conducted regarding sociodemographic characteristics including age (*t* test), sex distribution, education, social class, EMA compliance as well as negative affect score (survey design-based *F* test, Rao and Scott 1984).

Only EMA assessments with a time gap of less than 3 h in between each other were considered as truly consecutive EMA assessments. Hence, we also excluded between-day effects since night times were always longer than 3 h.

We investigated the relationship between *5*-*HTT*LPR/rs25531 genotype and the carry over effect of affect from one assessment to the next one, namely inertia (Kuppens et al. 2010), with regard to the negative affect scale using multilevel regression modeling to account for the multilevel structure of the data (assessments nested in subjects). In the models, the affect score at sampling time *t* was predicted by the affect score at sampling time *t*−1 (*t*−1 affect scale person mean-centered; Enders and Tofighi 2007). The estimated slope for prediction of *t* affect score by *t*−1 affect score represents the autocorrelation of the respective time series of affect ratings, which is a direct operationalization of inertia (Kuppens et al. 2010). In detail, we added the interaction between person mean-centered *t*−1 negative affect score and genotype information as predictors to a multilevel regression model to predict the negative affect score at time *t.* The regression coefficient of the interaction between *t*−1 affect score and genotype information indicates the difference in autocorrelation between the two genotype groups. Age, sex and type of day (week/weekend), the interaction of *t*−1 affect score and age as well as the interaction of *t*−1 affect score and sex were additionally entered as covariates in these analyzes, thereby adjusting for influence of age and sex on the slope of the *t*−1 negative affect score.

We first used a random intercept model with fixed slopes across subjects within genotype group and levels of covariates since we were only interested in comparing the overall mean autocorrelation within genotype groups against each other, not in investigating variance of autocorrelations across subjects within groups. Hence, we omitted a random slope model in the spirit of model parsimony at first. Since this model may not carry all important facets of the data, we then investigated a random slope model corresponding to van Roekel et al. (2018), where both the intercept and slope values were allowed to vary between all subjects and the intercepts and slopes are predicted by dummy-coded genotype information in the level 2 model part.

## Results

### Sample characteristics and assessment distribution

Sample characteristics and EMA compliance are shown in Table 1. For the present analysis, data of 877 participants were utilized. As participants sometimes quit the EMA assessment somewhere in between, different amounts of assessments were available for the analysis with respect to the measured variables (between 23.692 and 23.457). Genotype groups did not differ significantly concerning age, sex, education, social class, compliance and negative affect score (all *ps*_s_ > 0.05).

**Table 1:** Sample characteristics

	*5-HTT*LPR/rs25531		
	Total sample	L_A_L_A_	SS, SL_G_, L_G_L_G_, SL_A_, L_A_L_G_
*N*	877	289	588
Age [mean (SD)]	17.92 (2.35)	17.99 (2.32)	17.89 (2.36)
Sex (female %)	49.54	49.00	49.81
Education (%)			
Low	0.70	0.20	0.95
Middle	16.50	14.83	17.35
High	78.03	80.20	76.92
Other	4.77	4.77	4.77
Social class (%)			
Lowest	2.79	1.83	3.28
Lower middle	13.41	14.57	12.82
Middle	61.52	58.5	63.05
Upper middle	21.67	24.29	20.34
Upper	0.61	0.81	0.51
EMA compliance (%)	84.68	85.25	84.39
Negative affect (mean (SD))	17.02 (7.76)	17.28 (8.57)	16.89 (7.32)

Data were weighted to improve representativeness for sex, age and compliance, but frequencies are reported unweighted

*SD* standard deviation, Delete "NA" since "NA" not used in the table *NA* negative affect

### Association between 5-HTTLPR genotype and inertia regarding negative affect

**Fig. 1:**
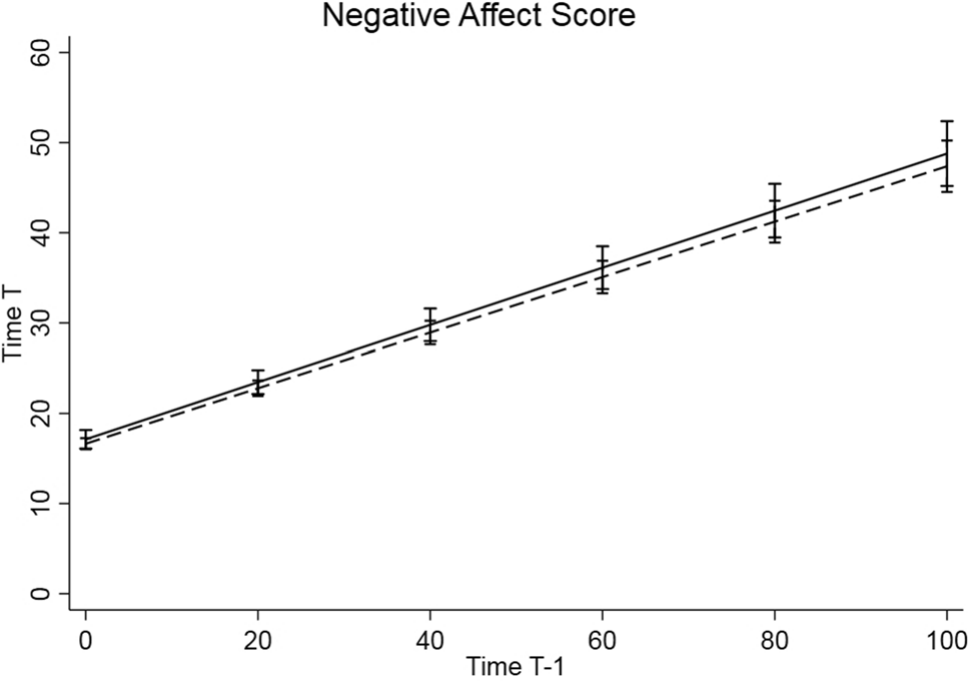
Illustration of inertia on Negative Affect Score based on the random slope model. *5-HTT*LPR/rs25531 genotype groups L_A_L_A_ vs. L_G_L_G_, SL_G_, SS, L_G_L_A_, SL_A_. Higher slopes represent higher inertia from one time point (Time *T*−1) to the next time point (Time *T*)

As shown in Table 2 and illustrated in Fig. 1, no association was found between *5-HTT*LPR/rs25531 genotype and inertia regarding negative affect in the parsimonious random intercept (*b* = 0.00, CI [− 0.05;0.05]) as well as in the random slope model (*b* = 0.01, CI [− 0.03; 0.05]).

**Table 2:** *5-HTT*LPR/rs25531 genotype and inertia regarding negative affect, results of multilevel regression [with *t* affect scale as response variable and *t*−1 affect scale, dummy-coded genotype information, and their interaction as predictors]

	Random intercept model			Random slope model		
	b	95% CI		b	95% CI	
Intercept negative affect						
Value	16.463	12.351	20.574	16.526	12.413	20.639
Genotype	0.485	− 0.708	1.678	0.492	− 0.702	1.686
Age	− 0.030	− 0.253	0.193	− 0.033	− 0.256	0.190
Female	2.130	1.064	3.197	2.138	1.072	3.204
Weekend	− 0.661	− 0.939	− 0.383	− 0.721	− 0.982	− 0.460
Slope negative affect						
Value	0.367	0.166	0.569	0.241	0.094	0.388
Genotype	0.000	− 0.050	0.050	0.009	− 0.033	0.051
Age	− 0.003	− 0.014	0.007	0.002	− 0.006	0.010
Female	0.082	0.030	0.134	0.058	0.016	0.101

Week/weekend is included as level 1 covariate, age and sex are included as level 2 covariates. The Intercept sections show the main effect of covariates on affect scores, which is the effect of the covariate if all other covariates were held at zero. The Slope sections show the effect of the covariate on the slope of the *t*−1 affect score, i.e. the regression coefficients of the interaction between covariate and person mean-centered *t*−1 affect score in predicting *t* affect score

*b* regression coefficient, *CI* confidence interval

### Post hoc analyses regarding associations between 5-HTTLPR genotype and inertia of specific emotion

**Fig. 2:**
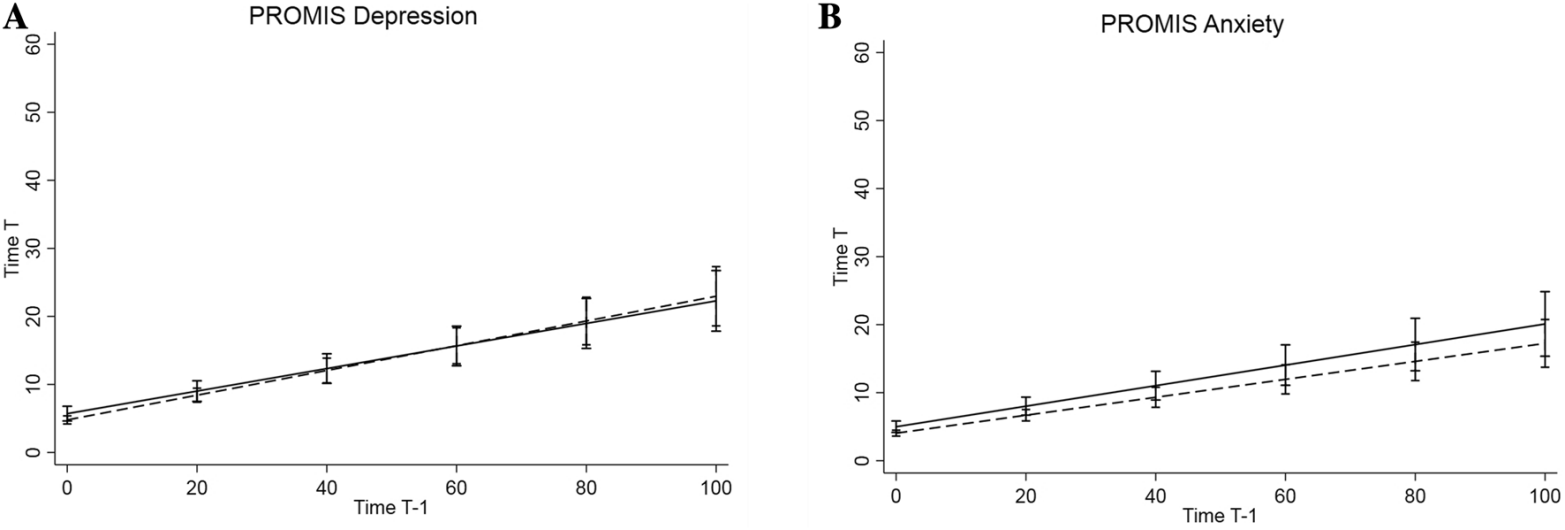
Illustration of inertia on **A** PROMIS Depression and **B** PROMIS Anxiety based on the random slope models. *5-HTT*LPR/rs25531 genotype groups L_A_L_A_ vs. L_G_L_G_, SL_G_, SS, L_G_L_A_, SL_A_. Higher slopes represent higher inertia from one time point (Time *T*−1) to the next time point (Time *T*)

Due to the unexpected lack of association between emotional inertia of negative affect and *5-HTT*LPR/rs25531, post hoc analyses were conducted to examine whether there is an association between *5-HTT*LPR/rs25531 and inertia of more specific emotional affect types. Specifically, the negative affect score was disentangled regarding inertia of depressive affect and inertia of anxious affect. This was based on the assumption by van Roekel et al. (2018), that emotional inertia not only constitutes a potential psychological pathway through which *5-HTT*LPR/rs25531 impacts on the risk for depression but also concerning the risk of more broadly affective disorders, i.e. also comprising anxiety disorders. In addition, EMA studies often combine different items and constructs to generate a negative affect score, as was the case in the study by van Roekel et al. (2018) as well as in the present study. This has been criticized by Dejonckheere et al. (2019) since the variation of these emotions in regard to arousal (Russell 2003), associated appraisals (Moors 2013) and behavioral tendencies (Frijda et al. 1989) was not taken into account. The results of the more realistic random slope model imposing less model assumptions serve as basis for interpretation. However, also the post hoc analyses, as shown in Table 3 and illustrated in Fig. 2, revealed no association of *5-HTT*LPR/rs25531 genotype and inertia of depression assessed by PROMIS-DEP in the two models (random intercept model: *b* = 0.01, CI [− 0.08, 0.10], random slope model: *b* = − 0.02, CI [− 0.07, 0.04]). A significant relationship was found, however, for *5-HTT*LPR*/*rs25531 genotype and inertia of anxiety assessed by PROMIS-ANX in the parsimonious random intercept model where equal slopes are assumed across subjects within genotype group and levels of covariates. L_A_L_A_ genotype predicted significantly higher levels of inertia concerning anxiety compared to SS, SL_G_, SL_A_, L_G_L_A_, L_G_L_G_ carriers (*b* = 0.09, CI [0.00, 0.17]). The association was reduced to near zero (*b* = 0.02, CI [− 0.04, 0.08]) and no longer significant in the more flexible random slope model where slopes are allowed to vary across all subjects. As mentioned above, the results of the random slope model are used for interpretation.

**Table 3:** *5-HTT*LPR/rs25531 Genotype and Inertia Regarding Depression and Anxiety, results of Multilevel Regression [with *t* affect scale as response variable and *t*−1 affect scale, dummy-coded genotype information, and their interaction as predictors]

	Random intercept model			Random slope model		
	b	95% CI		b	95% CI	
Intercept PROMIS depression						
Value	5.519	1.561	9.477	5.467	1.506	9.428
Genotype	0.877	− 0.344	2.098	0.927	− 0.297	2.151
Age	− 0.096	− 0.305	0.114	− 0.096	− 0.305	0.113
Female	2.254	1.185	3.324	2.321	1.253	3.390
Weekend	− 0.275	− 0.554	0.003	− 0.232	− 0.475	0.011
Slope PROMIS depression						
Value	0.579	0.186	0.971	0.263	0.040	0.487
Genotype	0.009	− 0.082	0.100	− 0.016	− 0.074	0.043
Age	− 0.023	− 0.044	− 0.002	− 0.008	− 0.021	0.004
Female	0.177	0.082	0.273	0.137	0.074	0.200
Intercept PROMIS anxiety						
Value	5.012	1.658	8.367	4.986	1.615	8.358
Genotype	0.922	− 0.016	1.859	0.954	0.013	1.895
Age	− 0.090	− 0.269	0.090	− 0.089	− 0.269	0.091
Female	1.509	0.710	2.309	1.546	0.744	2.348
Weekend	− 0.245	− 0.489	− 0.002	− 0.243	− 0.467	− 0.019
Slope PROMIS anxiety						
Value	0.171	− 0.149	0.491	0.153	− 0.078	0.383
Genotype	0.085	0.005	0.165	0.019	− 0.038	0.076
Age	− 0.003	− 0.019	0.014	− 0.003	− 0.016	0.009
Female	0.133	0.053	0.213	0.077	0.022	0.132

Week/weekend is included as level 1 covariate, age and sex are included as level 2 covariates. The Intercept sections show the main effect of covariates on affect scores, which is the effect of the covariate if all other covariates were held at zero. The Slope sections show the effect of the covariate on the slope of the *t*−1 affect score, i.e. the regression coefficients of the interaction between covariate and person mean-centered *t*−1 affect score in predicting *t* affect score

*b* regression coefficient, *CI* confidence interval

## Discussion

This EMA study conducted in a general population sample of adolescents and young adults did not confirm the previously by van Roekel et al. (2018) reported association between the *5-HTT*LPR*/*rs25531 S allele and inertia of negative affect. Thus, the results of the present study do not provide supporting evidence for the assumed psychological mechanism through which *5-HTT*LPR indirectly increases risk for depression as hypothesized by van Roekel et al. (2018). Also, disentangling negative affect by examining inertia separately for depressive affect and anxious affect revealed no robust associations with *5-HTT*LPR*/*rs25531.

The discrepancy of the present findings to the results found by van Roekel et al. (2018) could be due to various (methodological) reasons. First, rather than using a validated negative affect scale, the negative affect scores in both studies were generated by using different scales and a mix of items included in EMA, which are not identical. To combine different items and constructs to generate scales has been criticized by Dejonckheere et al. (2019) already, pointing out that this can lead to unreliable scales. This could further influence the quality of the scales and, therefore, the effects of inertia. However, the conducted post hoc analyses in the present study with regard to inertia of depressive and anxious affect revealed also no significant associations with *5-HTT*LPR/rs25531. Nevertheless, more research is needed using, depending on the research topic, validated affect scales, such as the Positive and Negative Affect Scale (Watson et al. 1988) or focus more on clear and single constructs to account for different arousals, appraisals and action tendencies.

Another reason for not being able to replicate the findings of van Roekel et al. (2018) might be the use of different assessment modalities. While the present study utilized a visual analogue scale (1–100) that is able to measure affect on a very detailed level, van Roekel et al. (2018) used a seven-point scale, where people might tend to answer in the same manner more often because of missing options which would match their answer exactly. Furthermore, EMA compliance was lower in the original paper, with about 76% compared to about 85% in the present analysis sample after excluding participants with compliance < 50%. Estimating the impact of higher missing rates is not possible, as no information was given on how missings were handled. The present study considered EMA assessments as truly consecutive with a time gap less than 3 h. Possible longer durations due to missings between assessments may affect the measurement of inertia. Additionally, differences in sample characteristics regarding age (van Roekel *M* = 14.2 years, SD = 0.5 vs. *M* = 17.4 years, SD = 2.3), or ethnicity (van Roekel: 97.1% born in Netherlands, 1.3% in non-European country, no information about Caucasian descent vs. only Caucasian descent by first generation) might account for the observed discrepancies between studies.

In addition, emotional inertia is a complex feature, expected to be influenced by genetic as well as environmental factors. In line, a recent study investigated the genetic and environmental contribution towards adolescent daily emotional inertia in a sample of adolescent twins who provided data in respect to their positive and negative emotions daily over a month. The authors showed that non-shared environmental influences play a significant role, whereas genetic influences appear to be of minor importance (Zheng and Asbury 2019). In line, meta-analytic evaluations of single-gene candidate studies point towards small to negligible effects of single candidate genes regarding psychological phenotypes (Border et al. 2019).

Further, emotional inertia was also found to be susceptible to stress (Koval and Kuppens 2012; Kuppens et al. 2010). Thus the discrepancies between the present findings and the findings by van Roekel et al. (2018) could be partly due to environmental influences not considered in the present study and the study by van Roekel et al. (2018).

Besides state measures like emotional inertia, it is important to note, that studies have also shown a link between *5-HTT*LPR or *5-HTT*LPR/rs25531, respectively, and anxiety sensitivity, partly in interaction with early trauma (Klauke et al. 2011), anxious temperament (Schiele et al. 2020), as well as trait anxiety (Gonda and Bagdy 2006; for review see Gottschalk and Domschke 2016). However, findings have been inconsistent; Osher et al. (2000) found a link between the S allele and anxiety-related traits covering harm avoidance and neuroticism in an adult sample, whereas the general association of *5-HTT*LPR and trait anxiety was not confirmed by a meta-analysis (Schinka et al. 2004) and in a recent study by Licht et al. (2020). In addition, *5-HTT*LPR has been linked to stress reactivity. Gunthert et al. (2007) showed that on days when college students experienced more intense stressors, S-allele carriers reported more feelings of anxiety, in comparison with L-allele carriers. Consequently, further investigations into the link between *5-HTT*LPR and emotional inertia should additionally consider trait measures of anxiety, stress reactivity or negative affect to clarify inconsistent findings.

Conclusions from the results of the present study should be drawn in light of some limitations as well as the fact that the present study did not allow for a one-to-one replication of the study by van Roekel et al. (2018) due to several reasons as detailed above. The limitations drawn from the present study concern first time slots where participants did not want to be interrupted by EMA, e.g. because of school or university duties. Thus, the representativeness of assessments of the daily life among the adolescents and young adults is limited. Additionally, it is worth noting that a qualified simplification of the computed multilevel model can alter results. However, the present study benefits from a detailed examination, investigating inertia not only regarding negative affect, but also exploring inertia of specific affects, i.e. depressive and anxious affect. A further advantage of our study is the large general population sample of adolescents in comparison to other studies investigating genetic effects applying EMA designs (Gunthert et al. 2007; van Roekel et al. 2018) as well as the study is close to a one-to-one replication.

To summarize, the present study could not replicate the previous finding of the *5*-*HTTLPR/rs25531* S allele being associated with inertia of negative affect (van Roekel et al. 2018). In addition, post hoc analysis revealed no significant relationship between *5-HTT*LPR/rs25531 genotype and inertia of depressive and anxious affect. Thus, further research is needed to clarify the association of *5-HTT*LPR/rs25531 and inertia regarding negative affect by using first, validated affect scales and second, considering environmental and contextual factors like stress).

## Appendix

See Table 4

**Table 4:** EMA items of the negative affect score

Construct	Reference	Item	Answers (0–100)
Negative affect score			
Depression	Patient-reported outcomes measurement information system (PROMIS emotional distress—depression—short-form, PROMIS-DEP)	Since the last beep I felt worthless	Never—sometimes—always
		Since the last beep I felt helpless	Never—sometimes—always
		Since the last beep I felt depressed	Never—sometimes—always
		Since the last beep I felt hopeless	Never—sometimes—always
Anxiety	Patient-reported outcomes measurement information system (PROMIS emotional distress—anxiety—short-form, PROMIS-ANX)	Since the last beep I felt fearful	Never—sometimes—always
		Since the last beep I found it hard to focus on anything other than my anxiety	Never—sometimes—always
		Since the last beep I felt worried	Never—sometimes—always
		Since the last beep I felt uneasy	Never—sometimes—always
Anger	Patient-reported outcomes measurement information system (PROMIS emotional distress—anger—short-form)	Since the last beep I was irritated more than people knew	Never—sometimes—always
		Since the last beep I felt angry	Never—sometimes—always
		Since the last beep I felt like I was ready to explode	Never—sometimes—always
		Since the last beep I was grouchy	Never—sometimes—always
		Since the last beep I felt annoyed	Never—sometimes—always
Experiential avoidance	Self-developed	Since the last beep I was upset and concerned about my feelings or thoughts	Never—sometimes—always
		Since the last beep I tried hard to get rid of my feelings or thoughts	Never—sometimes—always
Pessimism	Skala optimismus-pessimismus-2 (SOP2)	Currently I am pessimistic	Not at all—absolutely
Mood awake–sleepy	6-item short-form of the Multidimensional mental-state questionnaire (MDBF)	At the moment I feel…	Tired—awake
			Full of energy—shiftless
Negative thoughts	Self-developed	Since the last beep I thought about negative or unpleasant things	No negative thoughts—frequently negative thoughts
