# Correction to: *5-HTT* genotype and inertia of negative affect in adolescents and young adults from the general population

**DOI:** 10.1007/s00702-022-02489-2

**Published:** 2022-03-22

**Authors:** T. M. Ollmann, E. Seidl, J. Venz, L. Pieper, C. Voss, J. Hoyer, H. Kische, S. R. Poppenhäger, M. A. Schiele, K. Domschke, K. Beesdo-Baum

**Affiliations:** 1 Behavioral Epidemiology, Institute of Clinical Psychology and Psychotherapy, Technische Universität Dresden, Chemnitzer Str. 46, 01187 Dresden, Germany; 2 Center for Clinical Epidemiology and Longitudinal Studies (CELOS), Institute of Clinical Psychology and Psychotherapy, Technische Universität Dresden, Dresden, Germany; 3 Department of Psychiatry and Psychotherapy, Medical Center, Faculty of Medicine, University of Freiburg, Freiburg, Germany; 4 Center for Basics in NeuroModulation, Medical Faculty, University of Freiburg, Freiburg, Germany

## Correction to: Journal of Neural Transmission 10.1007/s00702-022-02459-8

The original version of this article unfortunately contained a mistake. Legend is missing under the Figs. 1 and 2.

The corrected Figs. 1 and 2 with caption are given in the following pages.

**Fig. 1:**
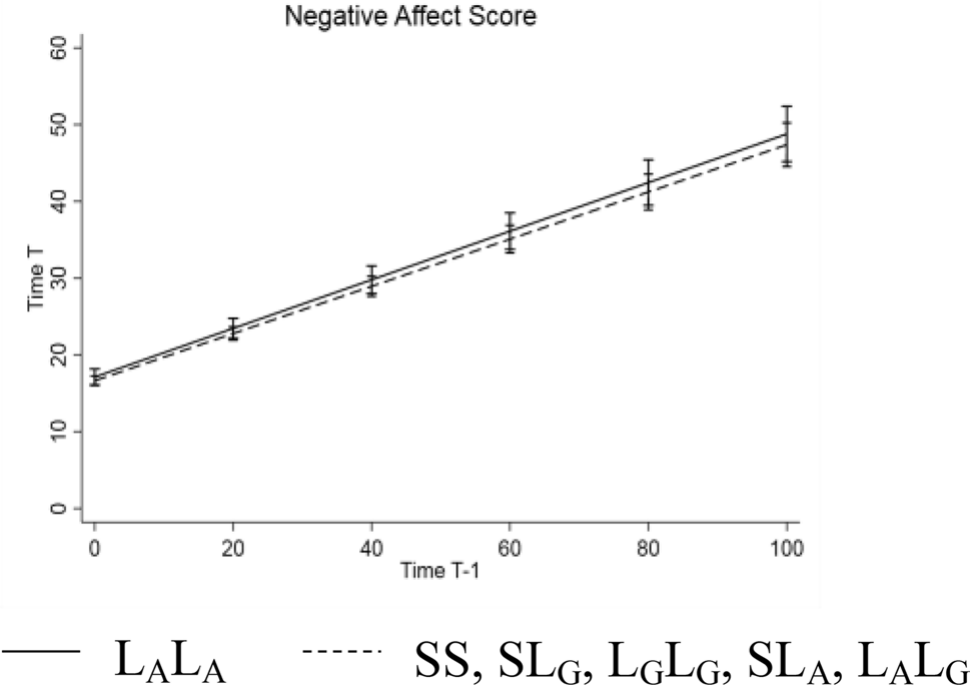
Illustration of inertia on Negative Affect Score based on the random slope model. *5-HTT*LPR/rs25531 genotype groups L_A_L_A_ (solid line) vs. L_G_L_G_, SL_G_, SS, L_G_L_A_, SL_A_ (dashed line). Higher slopes represent higher inertia from one time point (Time T − 1) to the next time point (Time T)

**Fig. 2:**
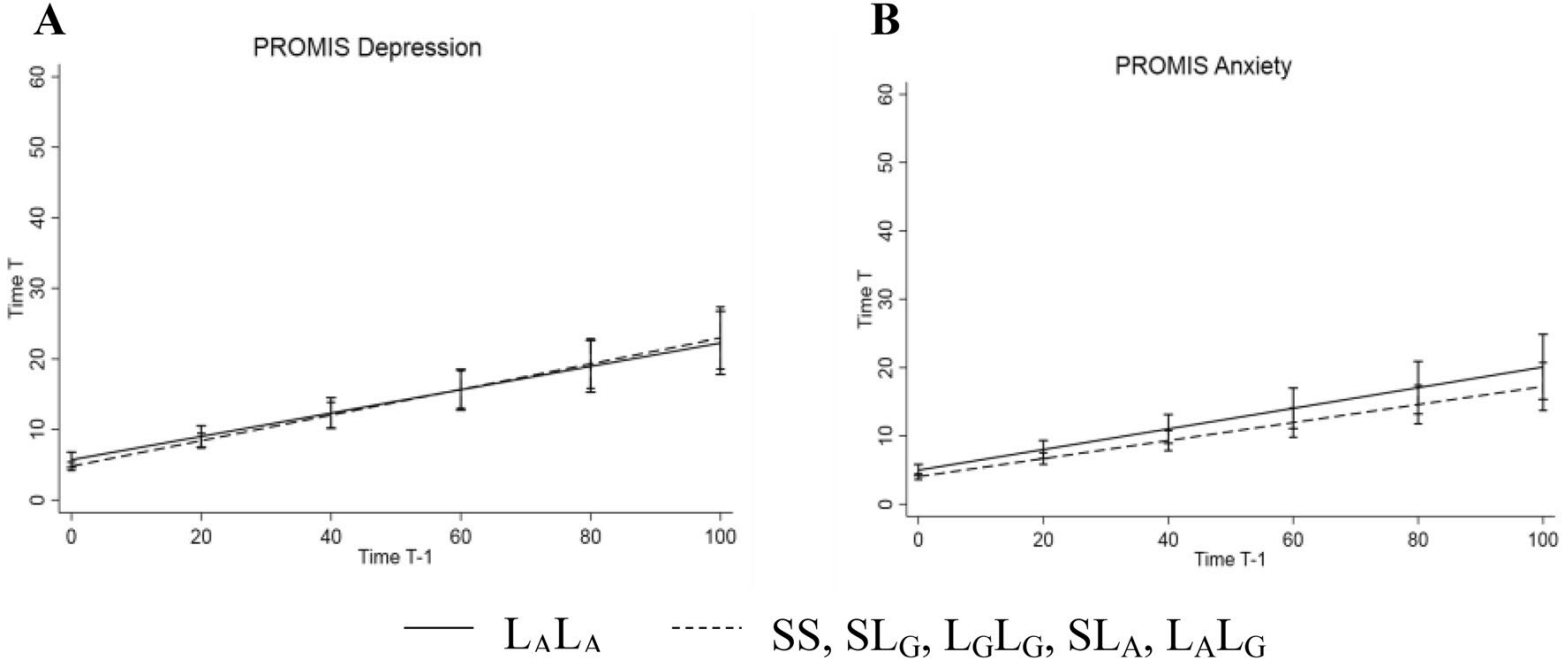
Illustration of inertia on **A** PROMIS Depression and **B** PROMIS Anxiety based on the random slope models. *5-HTT*LPR/rs25531 genotype groups L_A_L_A_ (solid line) vs. L_G_L_G_, SL_G_, SS, L_G_L_A_, SL_A_ (dashed line). Higher slopes represent higher inertia from one time point (Time T − 1) to the next time point (Time T)

